# A prospective study to evaluate the contribution of the pediatric appendicitis score in the decision process

**DOI:** 10.1186/s12887-024-04619-z

**Published:** 2024-02-19

**Authors:** Kevin Vevaud, Aymeric Dallocchio, Nathalie Dumoitier, Alban Laspougeas, Anaïs Labrunie, Alexis Belgacem, Laurent Fourcade, Quentin Ballouhey

**Affiliations:** 1 Service de chirurgie pédiatrique, Hôpital des Enfants, Hôpital Universitaire de Limoges, 8 Avenue Dominique Larrey, Limoges Cedex, 87042 France; 2 Département universitaire de médecine Générale, Faculté de médecine de Limoges, 2 rue du Docteur Marcland, Limoges Cedex, 87042 France; 3 Biostatistics and Research Methodology (CEBIMER), Limoges University Hospital, 2 rue du Docteur Marcland, Limoges Cedex, 87042 France

**Keywords:** Acute appendicitis, Children, Emergency, PAS score, Primary care

## Abstract

**Background:**

The objective of this study was to assess the likelihood of acute appendicitis (AA) in children presenting with abdominal symptoms at the emergency department (ED), based on their prior primary care (PC) consultation history.

**Methods:**

Between February and June 2021, we prospectively enrolled all children presenting at the ED with acute abdominal pain indicative of possible acute appendicitis (AA). Subsequently, they were categorized into three groups: those assessed by a PC physician (PG), those brought in by their family without a prior consultation (FG), and those admitted after a PC consultation without being assessed as such. The primary objective was to assess the probability of AA diagnosis using the Pediatric Appendicitis Score (PAS). Secondary objectives included analyzing PAS and C-reactive protein (CRP) levels based on the duration of pain and final diagnoses.

**Results:**

124 children were enrolled in the study (PG, *n* = 56; FG, *n* = 55; NG, *n* = 13). Among them, 29 patients (23.4%) were diagnosed with AA, with 13 cases (23.2%) from the PG and 14 cases (25.4%) from the FG. The mean PAS scores for AA cases from the PG and FG were 6.69 ± 1.75 and 7.57 ± 1.6, respectively, (*p* = 0.3340). Both PAS scores and CRP levels showed a significant correlation with AA severity. No cases of AA were observed with PAS scores < 4.

**Conclusions:**

There was no significant difference in PAS scores between patients addressed by PG and FG, even though PAS scores tended to be higher for patients with AA. We propose a new decision-making algorithm for PC practice, which incorporates inflammatory markers and pain duration.

**Trial registration:**

Institutional Ethics Committee registration number: 447-2021-103 (10/01/2021).

**Clinical trials registration number:**

ClinicalTrials.gov Identifier: NCT04885335 (Registered on 13/05/2021).

**Supplementary Information:**

The online version contains supplementary material available at 10.1186/s12887-024-04619-z.

## Background

Abdominal pain is a frequent complaint in pediatric emergency departments (EDs), with appendicectomy being the most commonly performed abdominal procedure worldwide [1, 2].

The lifetime risk of acute appendicitis (AA) is estimated at 7–8% [3]. Over the past few decades, there has been significant improvement in patient care, primarily attributed to advancements in imaging and biological assessment technologies. These advancements have enabled more accurate diagnosis, the exclusion of alternative conditions, and a reduction in unnecessary appendectomies. In France, the rate of hospitalizations for AA decreased by 32% between 1997 and 2006. In 2014, the incidence was 33 per 10,000 for individuals aged 10 to 19, with a peak occurring at 13 years of age [4]. Similar retrospective observations of hospitalization rates for AA have been documented in numerous European countries and the USA [5–7].

The diagnosis of AA based solely on a history of pain and typical clinical features is usually accurate in 70–80% of cases [8]. Mortality rates have significantly decreased in developed countries [2, 9]. However, diagnosing AA can present challenges, even for experienced pediatric surgeons, primarily due to its clinical variability and its ability to mimic other medical conditions [10]. In more than 30% of cases, the presentation may be atypical [11]. Additionally, assessing pain and conducting examinations in non-verbal children can be a complex task [12]. Depending on the study, initial misdiagnosis occurs in 28–57% of children under 12 years of age [13]. Misdiagnosis puts patients at risk of complications such as perforation and pelvic abscesses [14].

Therefore, in support of clinical decision-making, simple predictive scoring systems like the Alvarado score and the Pediatric Appendicitis Score (PAS) have been introduced to assess the likelihood of developing acute appendicitis and to aid in the determination ofwhich patients should undergo further investigation or observation [15, 16]. In 2002, Samuel et al. published the PAS score based on a cohort of children aged 4 to 15 years old. This score demonstrated sensitivity and specificity in the high 90th percentile [9]. Consequently, tt has since been considered a reliable tool for predicting AA [17–19].

Most previous studies have primarily examined the utility of the PAS score within ED populations, without considering prior consultations in primary care. Primary care physicians often serve as the initial assessors, typically lacking immediate access to extensive laboratory and imaging resources. Depending on their expertise, they are aware of the seriousness and potential morbidity associated with AA. The key question they must address in their practice is: “Should I refer this child for further investigations?”. The primary objective of this study was to compare the PAS scores of children presenting at the ED with clinical features suggestive of AA, taking into account whether or not they had a prior consultation in primary care. The secondary endpoint was to assess the potential of combined PAS scores and C-reactive protein (CRP) levels for diagnosing AA and alternative conditions.

## Methods

A prospective, observational, single-center study was conducted in the pediatric ED of our University Hospital from February to June 2021 (NCT04885335, first trial registration date 13/05/2021). Ethics approval for the study was obtained from the Institutional Ethics Committee of Limoges University Hospital (identification number 447-2021-103; first registration date 10/01/2021).

Patients aged 1 to 17 years, whose primary complaint was abdominal pain and who exhibited at least one of the items included in the PAS score, were recruited. Patients with abdominal pain lasting more than 7 days or a history of prior appendectomy were excluded. Additionally, patients referred to the ED for further evaluation within 7 days of an initial examination by a primary care physician were enrolled, despite the previous ED examination.

Among these patients, those displaying clinical indications suggestive of AA after an initial assessment by a senior doctor or a registrar in the ED were included and underwent blood sample collection. The enrolled children were categorized into three groups based on their pre-hospital management:

1. Family group (FG): Children brought in by their family without prior consultation.
2. Physician group (PG): Children referred by a primary care physician for further investigations.
3. Non-referred group (NG): Children admitted to the ED after a prior consultation in primary care, without a specific referral by the primary care physician for further investigations.

Data collected in the ED included the PAS score items, the final numerical score, as well as patient characteristics such as age, gender, history of a prior primary care consultation within 7 days, which blood cell (WBC) counts, and CRP values obtained either prior to or during ED hospitalization, duration of pain (< 12 h, 12–24 h, 24–48 h, and > 48 h), and the presence of abdominal guarding.

Adhering to the “do no harm” principle, blood tests were prescribed only when deemed necessary. As per the PAS score criteria, a WBC count exceeding 10,000/mm^3^ and a neutrophil count surpassing 7,500/mm^3^ were considered positive. Additionally, a CRP level exceeding 5 mg/L was considered positive. Ultrasonography served as the initial imaging examination, with computed tomography (CT) being reserved as a secondary option if ultrasonography did not yield conclusive results. CT scans were performed as a complement when complicated AA, such as an abdominal abscess, was suspected. All cases of mesenteric lymphadenitis (LM) were confirmed through positive imaging findings. AA diagnoses were conclusively established through perioperative macroscopic observations and categorized into one of two pathological groups:

1. Ancomplicated appendicitis: Inflammatory appendix and phlegmonous appendix.
2. Complicated appendicitis: Perforated appendix and appendicular peritonitis.

Definitive diagnoses were derived from reports issued by the ED, radiology, and pathology departments for AA cases. Patients and their characteristics were compared across, based on whether they had previously consulted in primary care and depending on the final diagnosis. A clinical PAS score (cPAS) was calculated, omitting and neutrophil counts, resulting in a total score of 8 points to reflect primary care conditions, as previously described [20].

Statistical analyses were conducted using SAS Enterprise Guide V7.1 software, with a significance threshold set at 0.05. was used to define statistical significance. Descriptive results for categorical variables are presented in numbers and percentages, while quantitative variables are expressed as means with their respective standard deviations (SD). The positive predictive value (PPV) and negative predictive value (NPV) of the PAS scores for diagnosing AA were also calculated, along with their corresponding 95% confidence intervals.

Comparisons of quantitative variables, such as PAS scores and CRP levels, based on binary variables, were assessed using the Mann–Whitney test. The analysis of these variables according to the diagnosis categories (“AA”, “mesenteric lymphadenitis (ML)”, or gastroenteritis (“GE”)) was conducted through an analysis of variance (ANOVA) test. Pairwise comparisons were adjusted using the Dunnett method. Additionally, the correlation between the PAS score and the CRP level was analyzed using the Spearman coefficient correlation test.

Comparisons of qualitative variables among groups (“FG”, PG”, or “NG”) or diagnoses were analyzed using chi-squared tests or Fisher’s tests as appropriate. The analysis of variations in PAS scores and CRP levels according to pain duration and diagnostic groups was performed using a general linear model. The explanatory variables considered in this analysis were the diagnoses (“AA,” “GE,” or “ML”), the duration of pain (“< 12 h,” “12–24 h,” “24–48 h,” and “> 48 h”), and the interactions between them.

## Results

### Patient characteristics

A total of 151 patients were initially enrolled over a span of four months; Subsequently, 27 patients were excluded, all of whom had been brought in by their family: 18 due to a history of prior appendectomy or abdominal pain persisting for more than 7 days, and 9 following a medical examination. Among the excluded cases, only one patient returned the next day and was subsequently included.

**Fig. 1:**
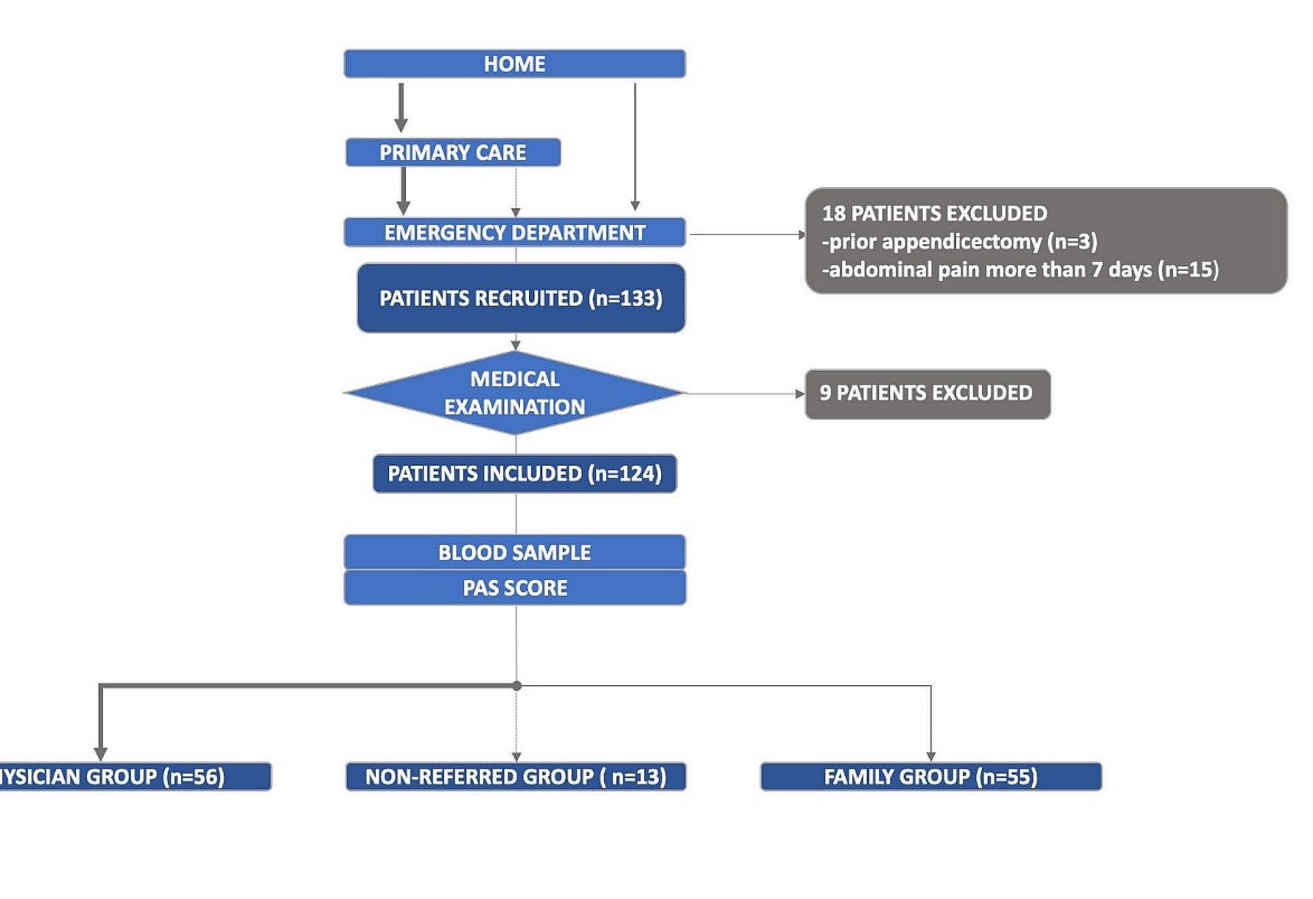
Study flowchart. AA = acute appendicitis; ED = emergency department; NA = non-addressed; NPC = with no prior consultation; PC = with prior consultation

Overall, 124 children were included in the three designated groups (FG *n* = 55, PG *n* = 56, and NG *n* = 13) *(*Fig. 1*)*. Notably, only three patients (all from the PG) had undergone blood tests prior to their ED admission.

**Fig. 2:**
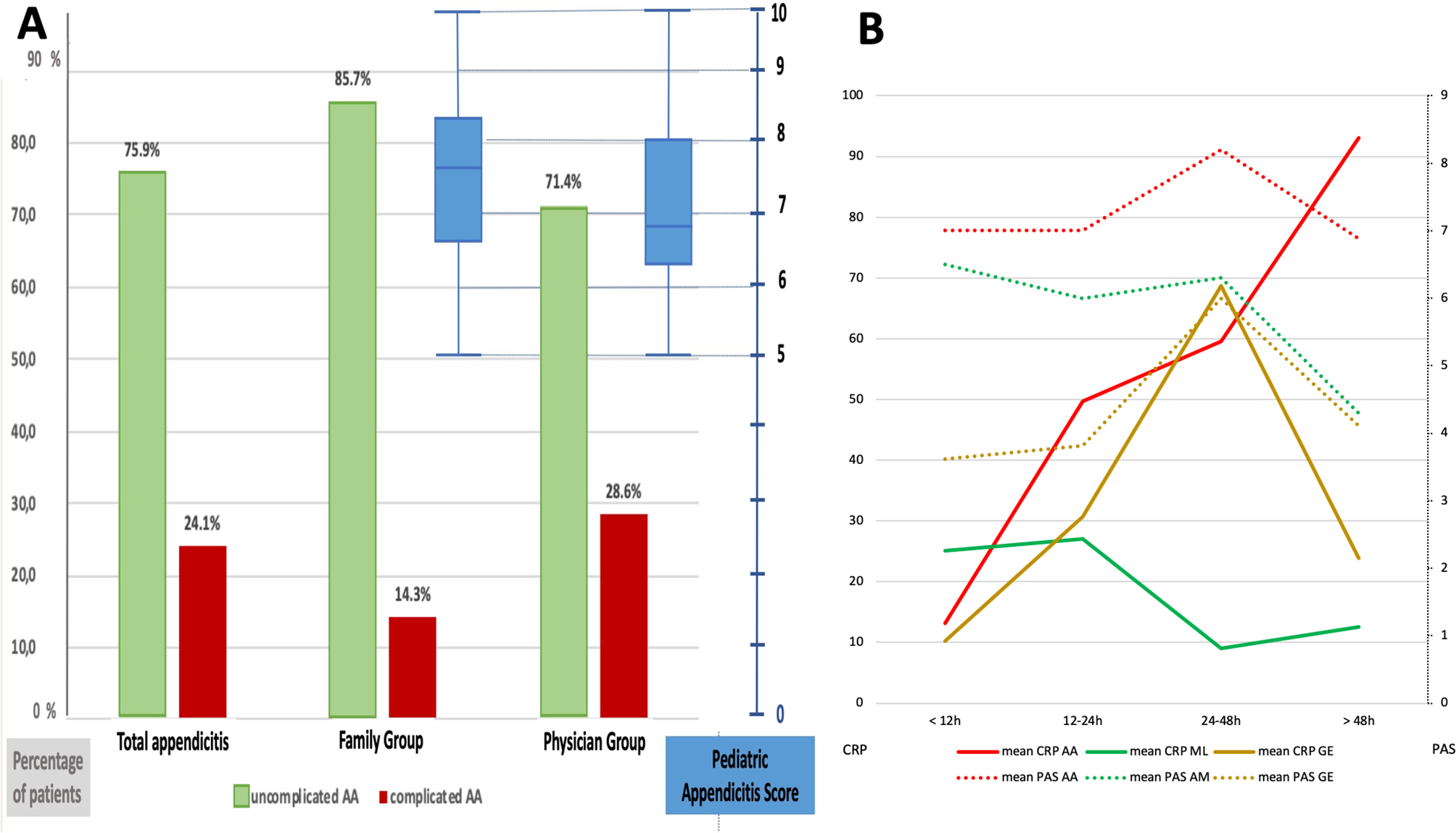
**A**. Distribution of patients depending on the severity of appendicitis. **B.** PAS scores and CRP levels in acute appendicitis (AA), mesenteric lymphadenitis (LM), and gastroenteritis (GE) according to pain duration

Out of the included patients, 30 children (24.2%) underwent laparoscopic surgery for AA, and 29 (23.4%) had AA macroscopically during surgery (Table 1). A male predominance was observed, with a sex ratio of 1.64. The mean age for boys was 10.05 ± 3.15 years, while for girls, it was 10.27 ± 3.41 years. Among the AA cases, 22 children had uncomplicated appendicitis (75.86%). Complicated appendicitis cases were most commonly observed in the PG (28.5%) (Fig. 2A*)*. There was a single perioperatively confirmed case categorized as ML based on ultrasonography findings. Consequently, the proportion of positive appendicectomies was similar between the FG (*n* = 14, 25.4%) and the PG (*n* = 13, 23.2%) (p-value = 0.7833). In the NG group (*n* = 13), there were only 2 cases of AA, 6 of ML, 1 of GE, 2 of constipation, and 2 cases with non-specific abdominal pain.

**Table 1:** PAS scores and characteristics of acute appendicitis, mesenteric lymphadenitis, gastro-enteritis and non-specific abdominal pain cases

	Patients (n)	Fever (%)	Anorexia (%)	N/V (%)	TRFI (%)	Hopping TRLQ (%)	Pain migration (%)	WBC count > 10,000/mm^3^ (%)	Neutrophils count > 7500/mm^3^ (%)	Mean PAS score (points) ± SD	Abdominal guarding (%)	Mean CRP (mg/L) ± SD
Total	124	33.9%	37.1%	66.1%	86.3%	45.2%	16.9%	35.5%	32.3%	4.84 ± 2.17	29.%	29 ± 45.06
Total FG	55	21.8%	30.9%	63.6%	81.8%	50.9%	16.4%	40%	36.4%	4.76 ± 2.40	29.1%	19.3 ± 38.33
Total PG	56	46.4%	44.6%	64.3%	91.1%	46.4%	14.3%	33.9%	30.4%	5.03 ± 1.92	30.4%	36.6 ± 47.19
Total NG	13	30.8%	30.8%	84.6%	84.6%	15.4%	30.8%	23.1%	23.1%	4.46 ± 2.22	23.1%	31.4 ± 51.04
Total AA	29	37.9%	62.1%	82.8%	96.6%	89.7%	20.7%	75.9%	69%	7.17 ± 1.69	82.8%	56.7 ± 55.89
AA FG	14	35.7%	57.1%	85.7%	92.9%	85.7%	35.7%	92.9%	92.9%	7.57 ± 1.60	71.4%	46.1 ± 58.78
AA PG	14	35.7%	64.3%	78.6%	100%	92.9%	7.1%	57.1%	42.9%	6.64 ± 1.75	92.9%	68.2 ± 54.94
AA NG	1	100%	100%	100%	100%	100%	0%	100%	100%	7.50 ± 2.12	100%	56 ± 55.15
Total ML	30	43.3%	36.6%	66.6%	100%	53.3%	30%	26.7%	23.3%	5.33 ± 1.54	16.7%	17 ± 29.55
ML FG	7	14.9%	42.9%	57.1%	100%	100%	14.3%	28.6%	14.3%	5.71 ± 1.11	28.6%	1.3 ± 0.48
ML PG	18	61.1%	33.3%	61.1%	100%	50%	33.3%	27.8%	27.8%	5.44 ± 1.50	16.7%	22.6 ± 31.77
ML NG	6	33.3%	33.3%	100%	100%	16.7%	33.3%	16.7%	16.7%	4.40 ± 2.07	0%	20 ± 38.74
Total GE	32	43.8%	34.4%	81.3%	71.9%	18.8%	9.4%	31.3%	31.6%	4.06 ± 1.74	6.3%	30.8 ± 40.67
GE FP	17	29.4%	23.5%	82.4%	70.6%	23.5%	5.9%	29.4%	29.4%	3.69 ± 1.78	11.8%	14.2 ± 13.98
GE PG	13	61.5%	46.1%	76.9%	76.9%	7.7%	7.7%	38.5%	38.5%	4.33 ± 1.68	0%	47.4 ± 53.02
GE NG	1	0%	0%	100%	0%	0%	100%	100%	0%	3 ± 0	0%	3 ± 0
Total NS	33	9.0%	18.1%	30.3%	69.7%	21.2%	12.1%	9.0%	6.0%	3.03 ± 1.23	15.1%	10.3 ± 37.1
NS FP	14	7.1%	14.2%	28.5%	78.5%	21.4%	14.2%	7.1%	7.1%	2.92 ± 1.49	14.2%	1.1 ± 0.56
NS PG	14	14.2%	28.5%	35.7%	85.7%	28.5%	7.1%	7.1%	7.1%	3.14 ± 1.02	21.4%	15.84 ± 15.24
NS NG	5	0%	0%	20%	0%	0%	20%	20%	0%	3 ± 0	0%	1.2 ± 0.34

AA = acute appendicitis; GE = gastro-enteritis; ML = mesenteric lymphadenitis; NS = non-specific abdominal pain group; FG = family group; PG = physician group; NG = non-addressed group; PAS = Pediatric Appendicitis Score; WBC = white blood cell; CRP = C-reactive protein; N/V = nausea or vomiting; TRFI = tenderness over the right fossa iliac; TRLQ = tenderness over the right lower quadrant

Uncomplicated appendicitis cases were predominantly associated with pain duration of less than 12 h (*n* = 9, 40.9%). No cases of complicated appendicitis were observed within the group with less than 12 h of pain. In contrast, complicated appendicitis cases were mostly identified after 48 h (*n* = 3, 42.8%). There were two initially misdiagnosed AA cases (6.9%).

The alternative diagnoses primarily consisted of GE (*n* = 32, 33.7%) and ML (*n* = 30, 31.6%). Additionally, there was a group of patients with non-specific abdominal pain (*n* = 33), encompassing cases of vague abdominal discomfort (*n* = 14, 14.7%), as well as other conditions such as urinary or gynecological pathologies (*n* = 10, 10.5%) and constipation (*n* = 9, 9.5%). Within this “non-specific group,” 9 sonographies and one abdominal computed tomography were performed, with no requirement for surgical intervention.

### PAS score

The PAS and cPAS scores are detailed in Table 2(Supplementary Material 1). Across all patients, the mean PAS score was 4.84 (± 2.17). There was no difference in the PAS score between the FG and the PG (4.74 ± 2.40 vs. 5.04 ± 1.9; *p* = 0.4249) *(*Fig. 2A*).* The PAS score was significantly higher in AA cases when compared to GE and ML cases (7.17 ± 1.69 vs. 4.09 ± 1.77 and 5.33 ± 1.54, respectively; *p* < 0.0001). The mean score for the non-specific abdominal pain group was 3.03 ± 1.98. Regarding AA cases, the mean PAS score was lower in the PG compared to the FG (6.69 ± 1.75 vs. 7.57± 1.6, respectively; *p* = 0.3340). Among the 16 children with PAS scores ≥ 8, 14 patients were diagnosed with AA, resulting in a positive predictive value (PPV) of 87.5%, with a 95% CI: [71.30–100.00]. There were no cases of appendicitis diagnosed for PAS scores < 4, yielding a negative predictive value (NPV) of 0.0%, 95% CI: [0.00; 0.00] *(*Table 3*)*.

**Table 2:** Distribution of the three main diagnoses according to the stratified PAS score and “clinical-PAS score”

	Patients (n)	AA (%)	ML (%)	GE (%)	NS (%)
PAS score < 4	37	0.00%	10.81%	35.13%	54%
PAS score 4–5	41	14.63%	31.70%	24.39%	29.2%
PAS score 6–7	30	30.00%	36.66%	30.00%	3.33%
PAS score ≥ 8	16	87.50%	12.50%	0.00%	0.00%
cPAS score < 4	47	4.25%	14.89%	34.04%	53.2%
cPAS score 4–5	47	19.14%	29.78%	29.78%	21.2%
cPAS score ≥ 6	30	60.00%	33.33%	6.66%	0.00%

PAS = pediatric appendicitis score (points); cPAS = clinical pediatric appendicitis score (points); AA = acute appendicitis;

ML = mesenteric lymphadenitis; GE = gastro-enteritis; NS = non-specific abdominal pain group

**Table 3:** Correlation between PAS scores and CRP levels in acute appendicitis, mesenteric lymphadenitis, gastro-enteritis, and non-specific abdominal pain group

**Total patients**	**PAS score** **stratum**	**Patients** (***n*** = **124**)	**Mean PAS score ± SD**	**Mean CRP ± SD**
	< 4	37	2.35 ± 0.63	3.26 ± 5.41
	4–5	41	4.46 ± 0.50	27.89 ± 44.79
	6–7	30	6.46 ± 0.50	32.86 ± 45.19
	≥ 8	16	8.62 ± 0.62	66.62 ± 56.92
**AA**	**PAS score** **stratum**	**Patients** (***n*** = **29**)	**Mean PAS score ± SD**	**Mean CRP ± SD**
	< 4	0	0	0
	4–5	6	4.66 ± 0.52	56.83 ± 36.58
	6–7	9	6.55 ± 0.53	36.66 ± 62.17
	≥ 8	14	8.64 ± 0.63	69.57 ± 58.13
**ML**	**PAS score** **stratum**	**Patients** (***n*** = **30**)	**Mean PAS score ± SD**	**Mean CRP ± SD**
	< 4	4	3 ± 0.00	3 ± 4
	4–5	13	4.61 ± 0.51	16.92 ± 30.83
	6–7	11	6.45 ± 0.52	17 ± 27.51
	≥ 8	2	8.5 ± 0.71	46 ± 60.81
**GE**	**PAS score** **stratum**	**Patients** (***n*** = **32**)	**Mean PAS score ± SD**	**Mean CRP ± SD**
	< 4	13	2.3 ± 0.63	6 ± 8.66
	4–5	10	4.4 ± 0.52	30.66 ± 50.28
	6–7	9	6.44 ± 0.53	50.11 ± 37.69
	≥ 8	0	0	0
**NS**	**PAS score** **stratum**	**Patients** (***n*** = **33**)	**Mean PAS score ± SD**	**Mean CRP ± SD**
	< 4	22	2.2 ± 0.64	2 ± 3.75
	4–5	10	4.2 ± 0.42	24.25 ± 61.43
	6–7	1	6	2
	≥ 8	0	0	0

PAS score = pediatric appendicitis score in points; CRP = C-reactive protein in mg/L; AA = acute appendicitis;

ML = mesenteric lymphadenitis; GE = gastro-enteritis; NS = non-specific abdominal pain group

### Correlation between PAS score, CRP level, and pain duration

The CRP values in AA cases were significantly higher than in ML cases (*p* = 0.0017), and they showed a tendency to be higher than in tGE cases (*p* = 0.0587). In AA cases, CRP levels did not show a significant correlation with PAS scores (*r* = 0.1581, *p* = 0.4124) and there was no significant association with PAS score stratification (*p* = 0.4014) (Table 3*)*. PAS scores and CRP levels were found to be correlated with the severity of AA (complicated vs. uncomplicated AA; *p* = 0.0005) (Supplementary material 2). When adjusting the mean PAS scores and CRP levels based on pain duration, they decreased after 48 h in the three primary diagnoses, except in AA cases where CRP values continued to rise *(*Fig. 2B*).*

## Discussion

Our study’s novelty lies in the comparison between patients referred by primary care physicians and those who self-referred to the ED. Primary care physicians can effectively use the PAS score to distinguish between children requiring further investigations and those who can be safely discharged home. In terms of pre-hospital management, no significant difference was observed in PAS scores between patients referred by a primary care physician and those brought in by their families. I It is important to note that our study had a relatively small sample size, which is unfortunate as it was conducted during the SARS-CoV-2 pandemic period. This period witnessed a significant reduction in AA presentations to EDs due to quarantine measures and government hygiene recommendations [21, 22]. Nevertheless, our findings underscore the utility of PAS scores, especially when combined with CRP values and pain duration.

### Absence of a significant difference in PAS scores between the PG and FG

We initially expected higher PAS scores and a greater incidence of AA in the PG, but our observations did not align with this expectation, as we observed similar numbers compared to the FG. Further analysis of individual PAS items provided more insights, revealing that fever and abdominal guarding were additional factors leading to ED hospitalization in AA cases. However, the analysis of PAS score items in the FG did not yield significant differences when compared to the PG. In AA cases, the mean PAS scores were higher in the PG compared to the FG. This trend may achieve statistical significance with a larger sample size. Families may prioritize clinical objective features such as pain intensity, vomiting or diarrhea leading to dehydration, and asthenia.

Notably, 21.4% of admitted children had PAS scores < 4, a trend observed similarly in both the FG and PG. Several factors may contribute to these observations, including logistical and health-related concerns. For instance, the ED is easily accessible, offers full-time consultations, and provides immediate access to necessary investigations, which may not be the case in general practitioner consulting rooms [23, 24]. Our data also indicated a preference for ED consultation when the pain duration was < 12 h, potentially associated with higher WBC counts.

### Higher CRP levels in the PG

CRP levels serve as a valuable diagnostic tool for assessing overall disease activity [25]. It can effectively rule out serious infections, with a high NPV [26] particularly when utilizing a threshold of 5 mg/L, or predict complicated appendicitis [8]. Our data analysis revealed a correlation between CRP levels and the severity of AA. CRP levels were significantly elevated in the PG compared to the FG across all three main diagnoses. Notably, cases of complicated appendicitis appeared to be more prevalent in patients referred by a primary care physician. Consistent with previous studies, we hypothesize that primary care physicians rely not only on the initial clinical examination but also on their clinical intuition or “gut feeling” that something may be amiss [27, 28].

### Differential kinetic profile between PAS and CRP

The decline in PAS scores after 48 h of pain could be attributed to variations in WBC and neutrophil counts. Indeed, the immediate response to inflammation typically involves an elevation of circulating leukocytes [29]. After 48 h, leukocytes, particularly neutrophils, tend to accumulate in inflamed tissues, resulting in lower levels in the serum [30, 31]. CRP levels tend to rise with bacterial infections and then decline exponentially within 18 to 20 h once the underlying stimulus diminishes [32]. Consequently, the kinetics of WBC and CRP may explain the changes observed in PAS scores and CRP levels around the 48-hour mark.

We identified only one study that assessed PAS scores at different time points. This prospective observational study of children with right lower quadrant pain [33] reported mean PAS scores similar to our results but did not observe a decrease in PAS scores for the AA or “non-appendicitis” groups. They concluded that a better cutoff for diagnosing AA was a score of 7 on days 1–2 and a score of 6 on day 3.

### Potentializing the PAS score with pain duration

**Fig. 3:**
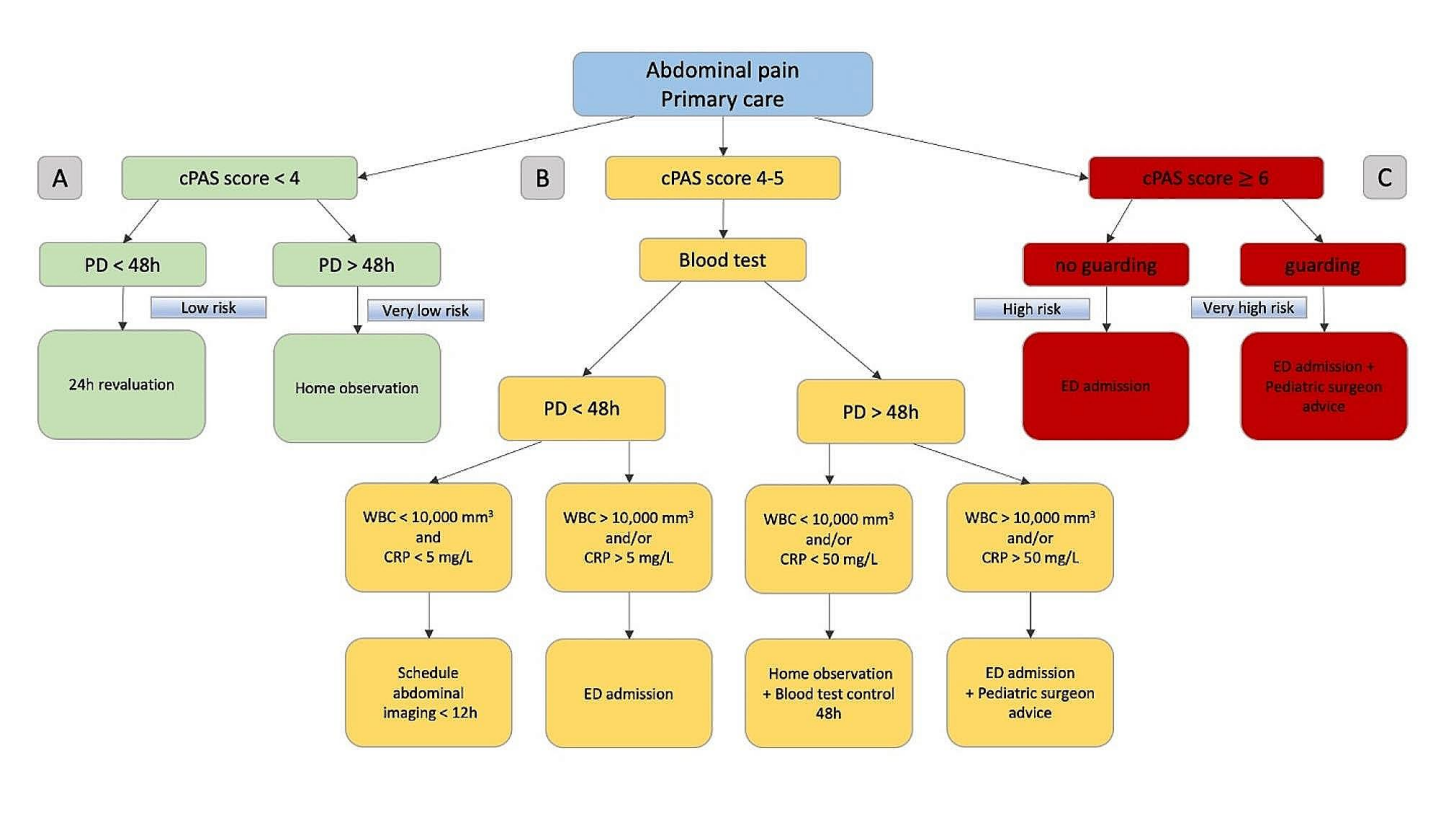
Proposal for the management of pediatric abdominal pain in primary care. Legend: We believe that diagnosing AA should primarily rely on clinical scoring. **A**: For cPAS < 4 with a duration of pain > 48 h, the risk is very low, and patients can be observed at home. If the pain onset is < 48 h, revaluation at 24 h is recommended. **B**: For intermediate cPAS scores from 4 to 5, inflammatory markers should be assessed. If the pain duration is < 48 h with normal inflammatory marker levels, scheduling imaging such as a ultrasound examination is recommended within 12 h. If one of the inflammatory markers is increased, however, ED admission is recommended. If the pain duration is > 48 h with normal inflammatory marker levels, home observation with a blood test at 48 h can be undertaken. Yet, if the inflammatory markers are increased, especially if the CRP level is above the 50 mg/L cutoff, timely admission to the ED is required. **C**: Thus, a cPAS score ≥ 6 would correspond to a high risk of AA and a very high risk if there is associated abdominal guarding. Referral to the ED is appropriate from the outset

The individual analysis of white blood cell (WBC) counts and CRP levels in the accuracy of AA diagnosis has produced conflicting results. Some studies found no significant differences in these parameters when comparing uncomplicated AA to non-AA cases [34, 35], often attributed to the limited sample size [36]. More recently, several studies have reported a strong correlation between elevated WBC counts and/or CRP levels with AA [37, 38], suggesting that analyzing these markers in combination can be valuable: elevated WBC counts may provide early indications of AA, while increased CRP levels may be more specific for perforation [8, 29, 36]. In addition, low CRP levels and WBC counts have shown high accuracy in ruling out AA diagnoses [38, 39]. In a retrospective cohort study of 1391 children [40] evaluating the time course of WBC, neutrophils, and CRP response between days 1 and 5 from initial right lower quadrant pain, the sensitivity of WBC and neutrophils decreased from day 1 to day 5 in AA cases (94.6% and 80.5% vs. 64.9% and 51.1%, respectively). Conversely, CRP exhibited increasing sensitivity, from 60.9% on day 1 to 97.9% on day 4. When combining all three parameters, sensitivity and negative predictive value were substantially improved to 99.7% and 98.7%, respectively, although the combined specificity remained low at 26.3%. In our study, no patient with abdominal pain evolving for more than 48 h, without fever, and normal WBC and CRP levels had AA, demonstrating an NPV of 100%. Taking into account existing literature and our findings, we propose a novel management model suited for primary care settings, incorporating the cPAS and PAS scores, inflammatory markers, and pain duration*(*Fig. 3*).* This algorithm is currently used within our facility after thorough discussions and approval by the general practitioners in our region. Its widespread adoption has fostered increased collaboration and communication between ED doctors and GPs since the completion of this study.

### Limitations

We had initially anticipated a larger number of children participating at the outset of the study. Extending the inclusion period for a longer duration to capture more cases would enhance the comparability between groups, particularly in the NG group. Additionally, it would provide more statistical significance to our PAS and cPAS score results.

Including patients over several months would be valuable, even though it is anticipated that there may be an increase in digestive and ENT disorders during the colder seasons. The exact number of children who were discharged following an initial consultation with their general practitioner is unknown. Additionally, it is unclear whether they were provided with a timely follow-up appointment or advised to visit the emergency department (ED) in case of worsening symptoms. In the PG group, PAS scores were assessed in the ED, and the scores may have been different if evaluated in primary care settings for the same patients. Lastly, pain intensity was not assessed, and this information could have been valuable for understanding the reasons behind ED visits.

In our study, no AA cases were observed for PAS < 4, and we consider this finding to potentially reflect real-life scenarios. It would be valuable to validate this observation through larger studies, aligning with the principles of our algorithm. However, it’s essential to note that we cannot generalize this observation at present. We acknowledge the potential utility of the correlation between PAS scores and CRP levels. However, it’s important to recognize that physicians are aware that CRP is an inflammatory marker and lacks specificity for any particular disease.

The decision algorithm presented in this study is a preliminary proposal, and it unquestionably necessitates further investigation and validation across diverse populations and geographical regions.

## Conclusion

This is the first prospective study to assess the risk of developing AA in children when consulting for abdominal pain in primary care. who initially consulted primary care for abdominal pain. Our findings indicate that children who consulted with a primary care physician before arriving at the ED did not exhibit a higher risk of developing AA compared to those who directly presented to the ED. Nearly 80% of these children received appropriate assessment using the PAS score. To minimize unnecessary ED visits, a coordinated approach between primary care and emergency care would be beneficial. We propose a new decision-making algorithm suitable for primary care settings, incorporating a revised PAS score, inflammatory markers, and pain duration. Its application in primary care conditions requires further study with larger sample sizes and longer inclusion periods, spanning an entire year, before it can be considered validated.

### Electronic supplementary material

Below is the link to the electronic supplementary material.

Supplementary Material 1

Supplementary Material 2

## Data Availability

the data and materials for this study are available upon request from the corresponding author at q.ballouhey@gmail.com.
