# Supplementary material for: A prospective study to evaluate the contribution of the pediatric appendicitis score in the decision process
Source: BMC Pediatr. 2024 Feb 19;24:131. doi: 10.1186/s12887-024-04619-z (PMC10875762; doi:10.1186/s12887-024-04619-z)
Supplement: Supplementary file 1 — Supplementary Material 1 [file 12887_2024_4619_MOESM1_ESM.docx]

**Table S1. Group distribution according to the PAS score stratification**

|  | **FG (n = 55) (%)** | **PG (n = 56) (%)** | **NG (n = 13) (%)** |
| --- | --- | --- | --- |
| PAS score < 4 | 32.72% | 21.42% | 53.84% |
| PAS score 4-7 | 52.72% | 67.85% | 30.76% |
| PAS score ≥ 8 | 14.54% | 10.71% | 15.38% |

PAS = pediatric appendicitis score (points); FG = family group; PG = physician group; NG = non-addressed group
