# Supplementary material for: A prospective study to evaluate the contribution of the pediatric appendicitis score in the decision process
Source: BMC Pediatr. 2024 Feb 19;24:131. doi: 10.1186/s12887-024-04619-z (PMC10875762; doi:10.1186/s12887-024-04619-z)
Supplement: Supplementary file 2 — Supplementary Material 2 [file 12887_2024_4619_MOESM2_ESM.docx]

**Table S2. Mean PAS score and CRP level according to the appendicitis severity**

|  | Mean PAS score (points) | Mean CRP (mg/L) |
| --- | --- | --- |
| Inflammatory appendix | 6.25 | 35.18 |
| Phlegmonous appendix | 8 | 45.33 |
| Perforated appendix | 9 | 106 |
| Appendicular peritonitis | 8.4 | 119.60 |

PAS score = pediatric appendicitis score; CRP = C-reactive protein in mg/L
